# The Synovial Sarcoma-Associated SYT-SSX2 Oncogene Antagonizes the Polycomb Complex Protein Bmi1

**DOI:** 10.1371/journal.pone.0005060

**Published:** 2009-04-01

**Authors:** Roy Barco, Christina B. Garcia, Josiane E. Eid

**Affiliations:** Department of Cancer Biology, Vanderbilt University Medical Center, Nashville, Tennessee, United States of America; Dresden University of Technology, Germany

## Abstract

This study demonstrates deregulation of polycomb activity by the synovial sarcoma-associated SYT-SSX2 oncogene, also known as SS18-SSX2. Synovial sarcoma is a soft tissue cancer associated with a recurrent t(X:18) translocation event that generates one of two fusion proteins, SYT-SSX1 or SYT-SSX2. The role of the translocation products in this disease is poorly understood. We present evidence that the SYT-SSX2 fusion protein interacts with the polycomb repressive complex and modulates its gene silencing activity. SYT-SSX2 causes destabilization of the polycomb subunit Bmi1, resulting in impairment of polycomb-associated histone H2A ubiquitination and reactivation of polycomb target genes. Silencing by polycomb complexes plays a vital role in numerous physiological processes. In recent years, numerous reports have implicated gain of polycomb silencing function in several cancers. This study provides evidence that, in the appropriate context, expression of the SYT-SSX2 oncogene leads to loss of polycomb function. It challenges the notion that cancer is solely associated with an increase in polycomb function and suggests that any imbalance in polycomb activity could drive the cell toward oncogenesis. These findings provide a mechanism by which the SYT-SSX2 chimera may contribute to synovial sarcoma pathogenesis.

## Introduction

The manipulation of chromatin organization is at the heart of a plethora of biological processes. Many proteins which modify the structure of chromatin during normal cellular events are often deregulated in disease processes, including cancer. One group of proteins involved in the stable and dynamic regulation of chromatin heritable over successive cellular divisions is the polycomb group (PcG) family of complexes [1]. PcG complexes are implicated in the repression of gene transcription through exquisite modulation of chromatin structure. They were originally identified in *Drosophila* as repressors of *Hox* genes for posterior body segmentation [2]. polycomb complexes are classically divided into Polycomb Repressive Complex 1 (PRC1) and Polycomb Repressive Complex 2 (PRC2) [1]. The PRC2 complex is comprised of the subunits EZH2, EED and SUZ12. PRC2 promotes gene silencing by associating with histone deacetylases and DNA-methyltransferase 1 [3], [4], and through methylation of histone H3 at lysine 27 (K27) [5], [6]. Methyl-H3K27 in turn serves as a binding platform for PRC1, composed mostly of PC2, HPH, Bmi1 and Ring1A/B. PRC1 compacts chromatin and hinders its accessibility to transcriptional activators [7]. Moreover, the complex between subunits Bmi1 and Ring1B (an E3 ligase) is implicated in the monoubiquitination of histone H2A at lysine 119 (K119) [8], a modification frequently detected in the promoters of polycomb target genes. Polycomb silencing plays an important role in cell fate determination, self-renewal in both embryonic and adult stem cells, as well as X-chromosome inactivation [1]. These processes result from polycomb suppression of key developmental pathways, including Wnt and Notch [9]–[11].

Recent evidence implicates deregulation of polycomb function in cancer promotion. A number of reports have described an increase in the expression of polycomb complex proteins in various malignancies; overexpression of Bmi1 in medulloblastoma [12], EZH2 in advanced prostate cancer [13] and SUZ12 in colon cancer [14]. These increases in protein level are thought to result in the aberrant silencing of tumor suppressor genes that would normally prevent carcinogenesis [15]. Conversely, several lines of evidence suggest that a decrease in polycomb function can favor tumor formation in the appropriate context. Downregulation of polycomb complex proteins has been described in certain tumors; these include reduced levels of Ring1B in germ cell tumors [16] and a decrease in Mel18 (polycomb RING Finger) in some breast cancers [17]. Additionally, many cancers, including leukemias and colon cancers, are associated with reactivation of *Hox* genes, the classical targets of polycomb silencing [18], [19]. More directly, one study determined that expression of a loss-of-function mutant of the PRC1 component PC2 resulted in the cellular transformation of fibroblasts [20]. Recently, a report demonstrated a reduction in histone H2A monoubiquitination- a polycomb-induced post-translational modification- in prostate cancer tumor samples [21]. Much like gain-of-polycomb function effects, these studies imply that perturbation of normal polycomb-mediated transcription repression is involved in some aspect of tumorigenesis. However, it remains to be seen whether any known oncogene can directly influence polycomb silencing in a negative fashion.

Synovial sarcoma is an aggressive soft tissue cancer that typically afflicts young adults. This disease is characterized by a persistent t(X;18)(p11;q11) translocation event that juxtaposes the *SYT* (SYnovial sarcoma Translocated) gene on chromosome 18 with an *SSX* gene (either *SSX1* or *SSX2*) on the X chromosome [22]. The resulting chimeric product, *SYT-SSX*, generates a fusion protein derived from both genes. The t(X;18)(p11;q11) rearrangement is detected in greater than 95% of synovial sarcoma tumors and is thought to play a crucial role in the genesis and progression of this cancer [22]. Previous studies have revealed that SYT-SSX1 possesses inherent transforming activity in both cell culture systems and nude mouse models [23]. The oncogenic capacity of SYT-SSX2 was also demonstrated in a transgenic mouse model whereby SYT-SSX2, expressed in *Myf5* lineage myoblasts, generated synovial sarcoma-like tumors with 100% penetrance [24]. Recently, it was demonstrated that SYT-SSX2 disrupted cellular positioning by remodeling the cytoskeleton and altering both cytoarchitecture and microtubule stability. The former was caused by activation of the ephrin pathway [25]. Despite these recent advances in the understanding of the biological consequences of the SYT-SSX chimeras, the specific mechanisms through which they execute these functions are unclear.

The SYT-SSX fusion product and its wildtype SYT and SSX counterparts are nuclear proteins believed to function as regulators of gene expression. Several studies have confirmed an interaction between the N-terminal domain of SYT (SNH; also present in SYT-SSX) with the SWI/SNF family of chromatin remodeling complexes [26]–[28] and the histone acetyltransferease p300 [29]. The specific contribution of SYT and SYT-SSX to chromatin modification and the biological consequences of these associations are currently unknown.

Recently, colocalization of both wildtype SSX and oncogenic SYT-SSX with the nuclear aggregates of polycomb complexes was reported [30]. A functional correlation between polycomb and the oncogene was also described [31]–[37]. These associations suggested that SYT-SSX likely modulates polycomb gene silencing function, an effect that could ultimately contribute to synovial sarcoma pathogenesis. However, a specific description of the functional consequence of SYT-SSX on polycomb repression is lacking. In this report, we demonstrate and further characterize the interaction between the SYT-SSX2 oncogene and the PRC1 polycomb complex. We also show that *de novo* expression of SYT-SSX2 induces a significant depletion of Bmi1 protein, an effect that is mediated at the post-translational level. Downregulation of Bmi1 was associated with attenuated monoubiquitination of histone H2A, a Bmi1-specific function. These events correlated with derepression of a variety of polycomb target genes involved in diverse cellular processes. These studies demonstrate for the first time polycomb antagonism as one possible mechanism by which SYT-SSX2 contributes to the epigenetic deregulation of normal transcription. They show negative regulation of polycomb function by a reputed oncogene and propose a novel paradigm regarding polycomb silencing and cancer-related proteins.

## Results

### SYT-SSX2 colocalizes and associates with polycomb components

**Figure 1:**
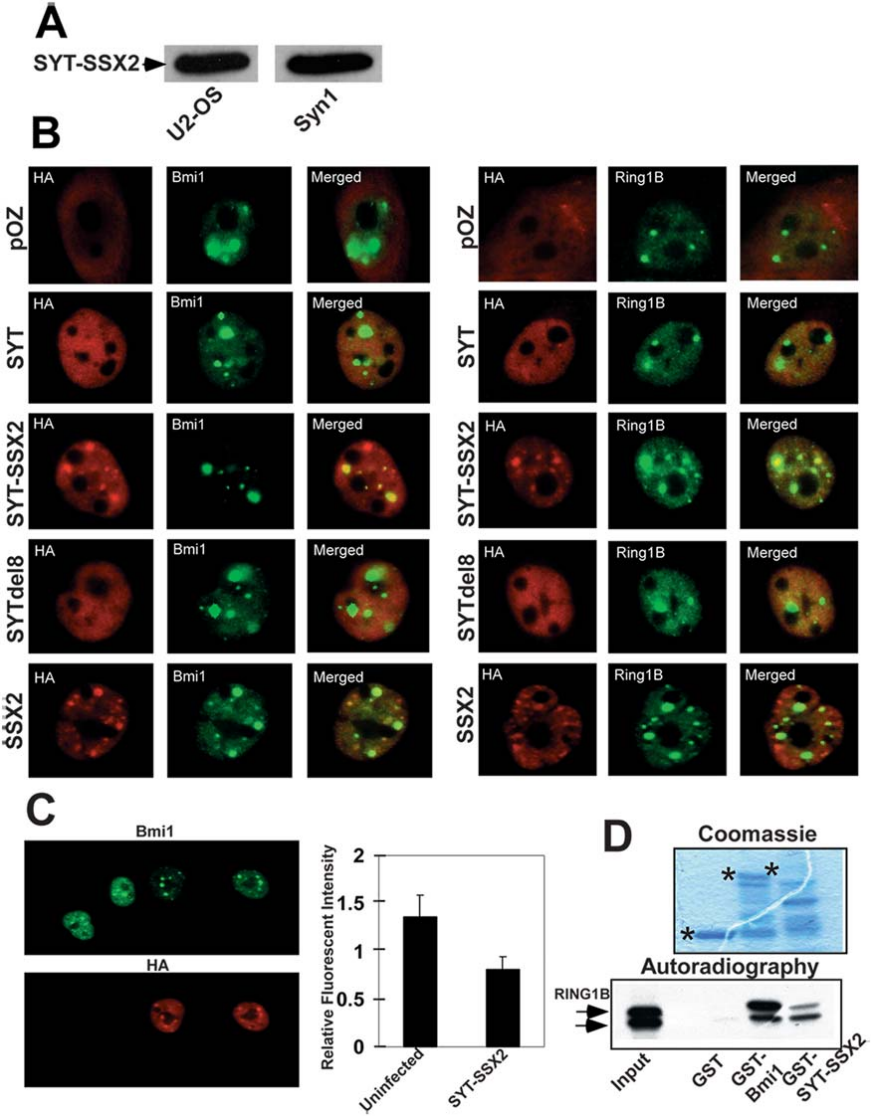
SYT-SSX2 associates with Polycomb complex proteins. (A) Equivalent protein levels of retrovirally expressed SYT-SSX2 (FLAG and HA-tagged) in U2-OS cells and in a primary human synovial sarcoma cell line (Syn1). 100 ug of cellular lysate-derived protein was loaded in each lane. SYT-SSX2 was visualized with the anti-FLAG tag antibody in U2-OS and with a SYT-specific antibody [SV11; 37] in Syn1 cells. (B) Colocalization of SYT-SSX2 with Bmi1 and Ring1B. Cells infected with pOZ viral vector, SYT, SYT-SSX2, SYTdel8, and SSX2 (all FLAG and HA-tagged), were analyzed by indirect immunofluorescence. Infected cDNAs (HA, red) and Bmi1 or Ring1B (green) were visualized individually and with merging of the two channels (merge). The cytoplasmic HA staining in pOZ-infected cells is due to the generation of an irrelevant FLAG/HA-tagged peptide by the vector. Image magnification was at 63×. (C) Decrease in Bmi1 fluorescence in SYT-SSX2 expressing cells. U2OS cells infected with SYT-SSX2 were analyzed by indirect immunofluorescence for SYT-SSX2 (HA; red) and Bmi (green). Image magnification was at 63×. Average fluorescence (20 cells per replicate; n = 3) of Bmi1 was compared between SYT-SSX2-infectants and uninfected cells using MetaMorph software and plotted. (D) *In vitro* binding of Ring1B to SYT-SSX2. Upper panel: GST, GST-Bmi1 and GST-SYT-SSX2 visualized by Coomassie staining. The asterisks (_*_) indicate full-length proteins. Lower panel: autoradiography of *in vitro*-translated (IVT) Ring1B bound to GST-Bmi1 (positive control) and GST-SYT-SSX2. Lane 1 represents 10% of input IVT Ring1B.

Previous reports have demonstrated colocalization between SYT-SSX and polycomb bodies, using Bmi1 and Ring1A as markers of polycomb complexes. These studies suggested that an interaction, whether direct or indirect, exists between these proteins. Similarly, using a retroviral system that allowed expression of SYT-SSX2 in U2OS cells (human osteosarcoma) at levels similar to those detected in primary synovial sarcoma cells (Syn1 cells- Figure 1A; in synovial sarcoma, *SYT-SSX2* expression is driven by the robustly active promoter of *SYT*), we were able to detect substantial colocalization of SYT-SSX2 with the polycomb protein Bmi1 (Figure 1B). U2OS cells were chosen for their shared mesenchymal origin with synovial sarcoma tumors. Overlapping distribution was most readily visualized in the Bmi1-containing polycomb bodies, where approximately 86% of these subnuclear structures were co-occupied by SYT-SSX2 aggregates. Colocalization of SYT-SSX2 was also observed with the polycomb complex component Ring1B (Figure 1B), where co-aggregation was slightly higher (93%). the SYT portion of the chimera alone (SYTdel8) failed to display significant colocalization (Figure 1B), indicating that targeting to polycomb bodies required the presence of the SSX2 component in the SYT-SSX2 fusion protein. Further analysis confirmed that when expressed alone, the SSX2 component of the chimera is capable of localizing to polycomb bodies. SSX2 was detected in 78% of Bmi1- and 75% of Ring1B-containing foci (Figure 1B). These indirect immunofluorescence experiments, in accordance with others, suggested that SYT-SSX2 might physically interact with polycomb complexes. We next wanted to determine whether a direct interaction between SYT-SSX2 and Bmi1 or Ring1B was responsible for targeting SYT-SSX2 to polycomb foci. Using *in vitro* GST pull-down assays, we detected specific binding between SYT-SSX2 and Ring1B (Figure 1D) but failed to demonstrate a similar interaction with the Bmi1 protein (Figure S1). These results suggest that SYT-SSX2 colocalization with the Bmi1/Ring1B complex in polycomb bodies is likely mediated by its direct binding to Ring1B. Further characterization of the SYT-SSX2/Ring1B association is underway.

### Loss of Bmi1 immunoreactivity in SYT-SSX2-infectants

**Figure 2:**
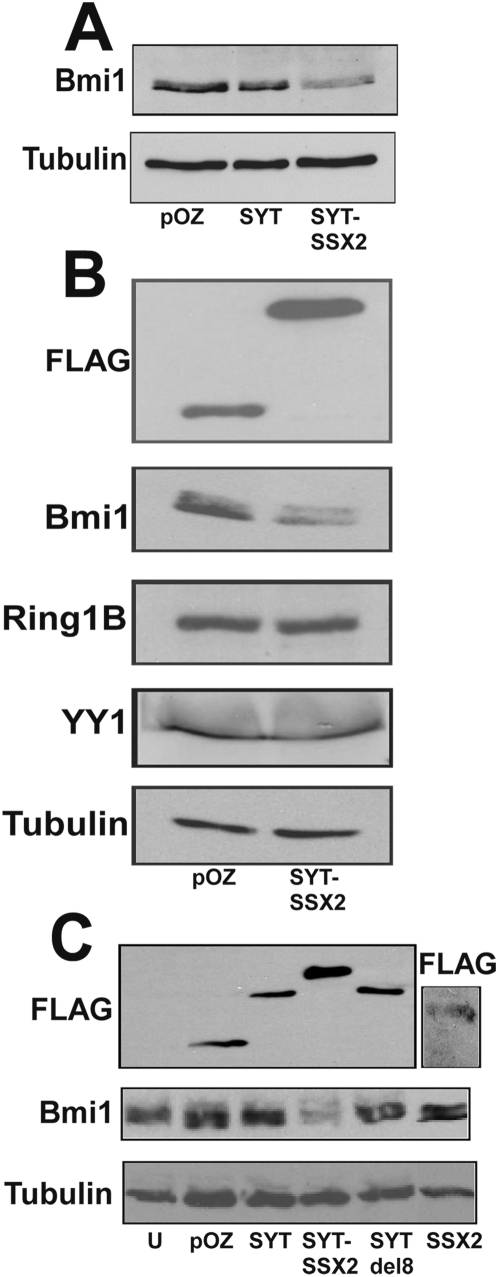
Loss of Bmi1 immunoreactivity following de novo expression of SYT-SSX2. (A) Extracts derived from pOZ vector, SYT and SYT-SSX2 cells were analyzed by Western blotting for levels of Bmi1 and alpha-tubulin (loading control). (B) Loss of immunoreactivity is specific to Bmi1. pOZ and SYT-SSX2-infectants were lysed and immunoblotted for Bmi1, Ring1B, YY1, FLAG (to detect ectopically expressed proteins) and alpha-tubulin. (C) The decrease in Bmi1 signal is a specific function of SYT-SSX2 chimera. Extracts from uninfected cells, pOZ, SYT, SYT-SSX2, SYTdel8 and SSX2 were immunoblotted for Bmi1, FLAG-tagged ectopically expressed proteins and alpha-tubulin. The FLAG-tagged protein visualized in pOZ-infected cells is due to the generation of an irrelevant FLAG/HA-tagged peptide by the vector. The molecular weight of the SSX2 domain is ∼10 Kd. For its detection we ran the SSX2 lysates on a separate 18% SDS-PAGE system. The remaining lysates were resolved on a 10% SDS-PAGE. U: uninfected cells.

During the Bmi1/SYT-SSX2 colocalization experiments, we observed a substantial reduction in Bmi1, but not Ring1B, fluoresecent intensity in SYT-SSX2-expressing cells (Figure 1 B and C). This reduction in Bmi1 fluorescence was quantified by MetaMorph software as approximately 50% of wildtype levels (Figure 1C). There was an apparent loss of not only the Bmi1 pool distributed diffusely throughout the nucleoplasm, but in the Bmi1 pool present in polycomb bodies as well. No change in the Bmi1 nuclear pool was observed in pOZ vector control and wildtype SYT-infectants. We also noted that localization of SSX2 in the Bmi1- and Ring1B-bearing foci was not accompanied by an attenuation of the Bmi1 signal (Figure 1B). Interestingly, the specific loss of Bmi1 immunofluorescence associated with SYT-SSX2 expression was consistently accompanied by an apparent decrease of Bmi1 protein levels on immunoblots of SYT-SSX2-transduced cellular extracts, using the same Bmi1 monoclonal antibody (Figure 2A). Conversely, the levels of other polycomb complex subunits, such as Ring1B and YY1 in SYT-SSX2 infectants were unchanged (Figure 2B), highlighting the specificity of this depletion effect for Bmi1. Consistent with the Bmi1 fluorescence signal, the singular expression of the SYT or the SSX2 fragment present in the chimera (SYTdel8, SSX2) did not affect Bmi1 protein levels (Figure 2C). Altogether, these results suggest that the SSX2 domain targets SYT-SSX2 to polycomb foci. However, its fusion to SYT is required to cause diminution of Bmi1 cellular levels. This effect is therefore specific to the SYT-SSX2 oncogene.

**Figure 3:**
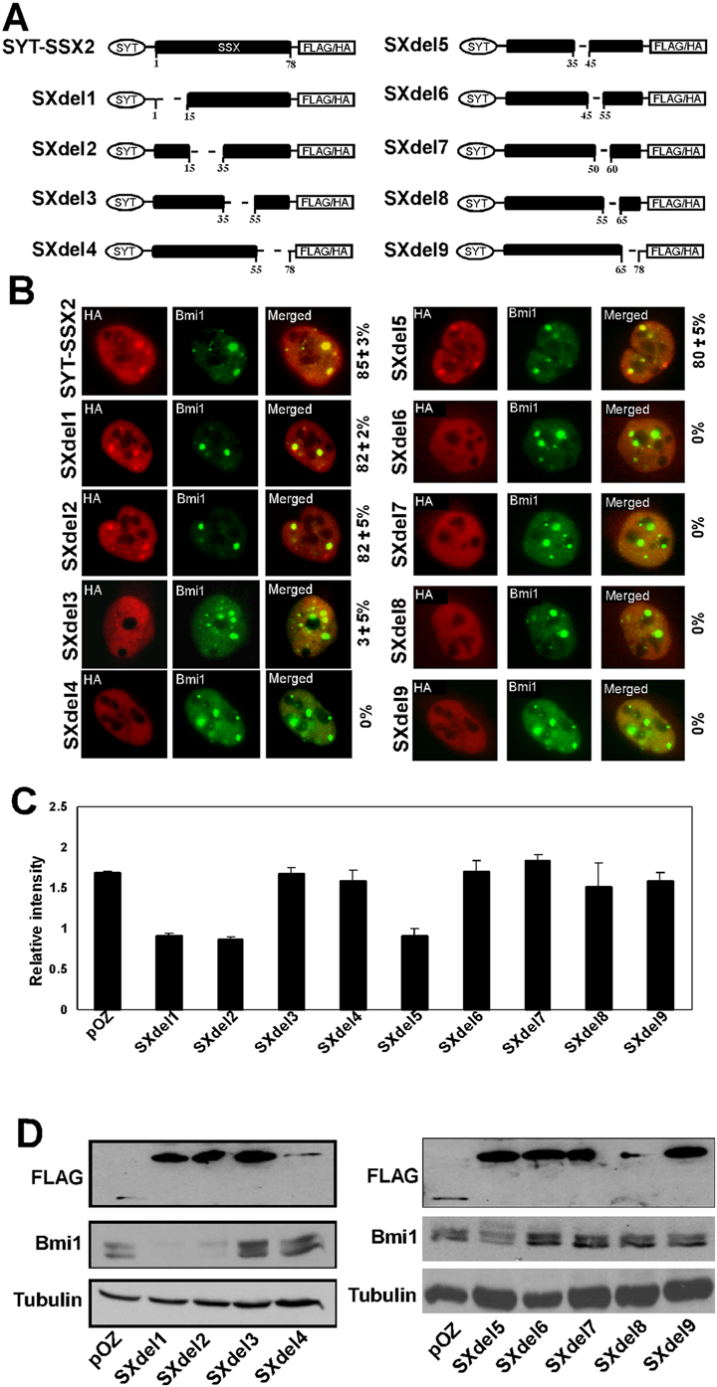
Mapping of the Polycomb association region on SYT-SSX2. (A) Schematic of wildtype SYT-SSX2 and the truncation mutants generated within the SSX2 region. (B) Immunofluorescent colocalization studies of the SYT-SSX2 truncation mutants (HA, red) and Bmi1 (green), with merging of the two channels displayed as well. Also indicated is the average percentage of SYT-SSX2 (or mutant) proteins aggregating with polycomb bodies (a minimum of 100 polycomb foci counted per replicate; n = 3). Image magnification was at 63×. (C) Plot of Bmi1 fluorescence in U2OS cells expressing the SXdel mutants and the POZ control vector. Average fluorescence (20 cells per replicate; n = 3) of Bmi1 was compared between POZ- and SXdel-infectants. The Bmi1 fluorescence in the POZ nuclei was consistently equivalent to that of uninfected cells. Data analysis was performed using MetaMorph software. (D) Loss of Bmi1 immunoreactivity correlates with association of SYT-SSX2 to polycomb complexes. Western blotting of cells infected with indicated FLAG-tagged proteins using Bmi1, FLAG and alpha-tubulin-specific antibodies.

To gain a better insight into the decreased immunoreactivity of Bmi1 in SYT-SSX2-infectants, we generated a series of truncation mutants of SYT-SSX2 and expressed them in U2OS cells. The first set of deletions, named SXdel1-del4 (Figure 3A), contained truncations between 15–23 amino acids in length. Mutants SXdel1 and SXdel2, corresponding to deletion of amino acids 1–35 of the translocated SSX2 domain (Figure 3A), retained their ability to colocalize with polycomb complexes (Figure 3B). Conversely, mutants SXdel3 and SXdel4, which lack amino acids 35–78, lost their association with polycomb bodies. This suggested that the C-terminal 44 amino acids contain the region essential for localization of SYT-SSX2 to polycomb complexes. We further refined the mapping of SYT-SSX2- polycomb association by creating smaller truncations within the C-terminal 44 amino acids of SSX2; these mutants were designated SXdel5-del9 (Figure 3A). Of these mutants, only SXdel5 retained its polycomb colocalization ability (Figure 3B), suggesting that the C-terminal 34 amino acids of SYT-SSX2 contain the polycomb association motif. This C-terminal stretch lacks homology to any known protein-protein interaction domains and represents a novel interface for association with polycomb components or their interacting proteins. Like its SXdel4 counterpart, mutant SXdel8 with truncated amino acids 55–65 expressed poorly (Figure 3D), suggesting that region maintains proper stability of SYT-SSX2. As anticipated, expression of the SYT-SSX2 mutants that co-localized with polycomb complexes (SXdel1, del2, and del5) induced depletion of endogenous Bmi1 protein (Fig. 3B and D). These same three mutants also exhibited a decreased Bmi1 fluorescence in their nuclei (Fig. 3C). The remaining SYT-SSX2 mutants that failed to be targeted to polycomb bodies had no effect on Bmi1 cellular levels. Taken together, these experiments suggest that SYT-SSX2 requires close association with polycomb complexes in order to induce a decrease in Bmi1 immunoreactivity.

### Loss of Bmi1 immunoreactivity is due to a decrease in Bmi1 protein stability

**Figure 4:**
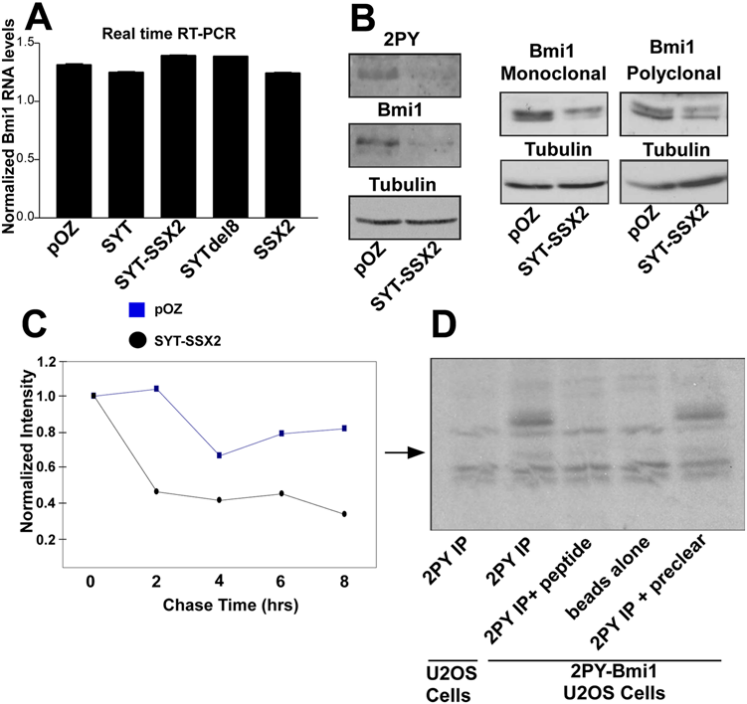
Loss of Bmi1 immunoreactivity in SYT-SSX2-infected cells results from depletion of the Bmi1 protein. (A) Real time RT-PCR of Bmi1 in U2OS lysates expressing the indicated proteins. (B) Left panel: overexpressed polyoma epitope-tagged Bmi1 (2PY-Bmi1) was infected with either SYT-SSX2 or pOZ backbone. Cell lysates were immunoblotted with Bmi1 antibody, then stripped and reprobed with an anti polyoma tag antibody. Immunoblotting for alpha-tubulin was used as a loading control. Right panel: cell lysates of pOZ and SYT-SSX2-infected cells were immunoblotted for either mouse monoclonal or rabbit polyclonal Bmi1 antibody. Immunoblotting for alpha-tubulin was used as a loading control. (C) Densitometry plotting of pulse-chase analysis of 2PY-Bmi1 in pOZ and SYT-SSX2-infected cells. 2PY-Bmi1-expressing cells infected with pOZ and SYT-SSX2 were labeled for 1 hr. with S^35^–labeled Methionine and Cysteine. Labeled cells were chased at the indicated timepoints and immunoprecipitated with a polyoma tag antibody. Immunoprecipitated 2PY-Bmi1 band intensities were quantitated by densitometry and plotted with pOZ and SYT-SSX2 experiments indicated. Pulse-chase experiment was successfully replicated (n = 2). (D) Immunoprecipitation studies demonstrating the specificity of the immunoprecipitated 2PY-Bmi1 band used in pulse-chase analysis. Negative controls using polyoma peptide blocking (lane 3), no antibody (lane 4) and naïve U2OS cells (lane 1) are indicated.

Several explanations can account for the decrease in Bmi1 signal intensity in SYT-SSX2 infectants observed on cellular extracts immunoblots and by indirect immunofluorescence; these include a decrease in the transcription of Bmi1, a decreased half-life of Bmi1 protein and a post-translational modification that impairs epitope recognition by the Bmi1 monoclonal antibody used in our studies. We did not detect changes in Bmi1 mRNA levels following transduction of SYT-SSX2 by both relative RT-PCR (Figure S2) and real time RT-PCR (Figure 4A), arguing against transcriptional inhibition as the mechanism for Bmi1 depleted signal. We then carried out three different studies to ascertain whether the lack of Bmi1 immunoreactivity resulted from decreased Bmi1 protein stability or a *de novo* Bmi1 modification. In the first experiment, U2OS cells were transduced with Bmi1 cDNA that was fused to two copies of the polyoma epitope tag (2PY-Bmi1). These cells were re-infected with either pOZ retroviral backbone or SYT-SSX2 cDNAs and lysed 48 hours later. Immunoblotting for both the polyoma tag (2PY) and Bmi1 revealed signal depletion in the SYT-SSX2-infectants using both antibodies (Figure 4B). This argues against a Bmi1 post-translational modification, since an epitope-masking modification should not interfere with the ability of the polyoma tag antibody to recognize tagged Bmi1. In another experiment, lysates from either pOZ or SYT-SSX2-infected cells were blotted for endogenous Bmi1 using both a monoclonal and a polyclonal Bmi1-specific antibodies. Both antibodies revealed decreased Bmi1 levels in lysates from SYT-SSX2-transduced cells (Figure 4B), again negating the possibility of epitope-masking by a Bmi1 post-translational modification. Moreover, mass-spectrometry analysis of Bmi1 present in SYT-SSX2 cellular lysates failed to detect phosphorylated, acetylated or methylated residues (data not shown). In the final experiment, the half-life of 2PY-Bmi1 was measured using pulse-chase experiments in both pOZ and SYT-SSX2 infectants. Not surprisingly, Bmi1 in SYT-SSX2-expressing cells exhibited accelerated degradation when compared to pOZ control (Fig. 4C). We confirmed the correct identity of the immunoprecipitated 2PY-Bmi1 by peptide competition studies (Figure 4D). Regression analysis on the pulse chase curves revealed a half-life of Bmi1 at 13.1 hours in pOZ vector-infected cells and 4.9 hours in SYT-SSX2 expressants. These studies suggest that decrease in Bmi1 protein stability is the contributing factor to Bmi1 signal depletion in SYT-SSX2-infected cells. The decision to use the 2PY-Bmi1 in the pulse-chase experiments was based on the fact that expressed 2PY-Bmi1 exhibited similar behavior to that of endogenous Bmi1 in SYT-SSX2 expressants and on the high immunoprecipitating efficiency of the 2PY antibody (Figure 4B).

### Expression of the SYT-SSX2 compromises the integrity of Bmi1/Ring1B complex and impairs its function in histone H2A ubiquitination

**Figure 5:**
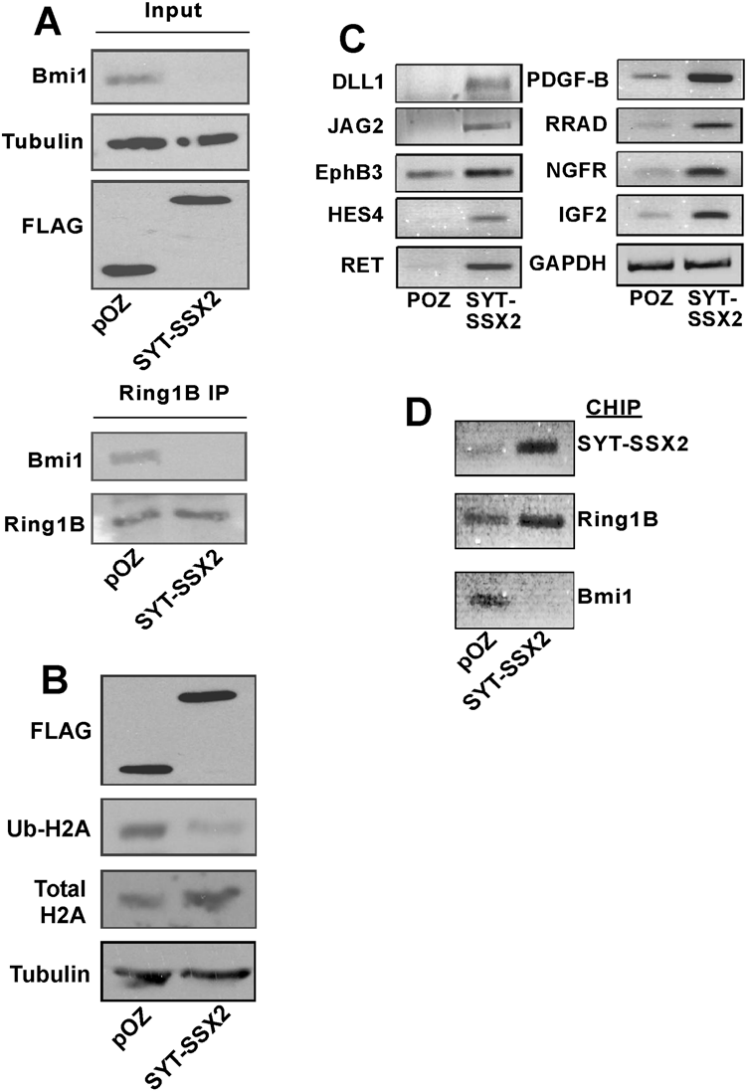
Functional antagonism of Bmi1 in SYT-SSX2-expressing cells. (A) Lower panel: the Ring1B-interacting Bmi1 population is depleted in SYT-SSX2-expressing cells. pOZ and SYT-SSX2 infected cell extracts were immunoprecipitated (IP) with a Ring1B antibody. Immunoprecipitates were immunoblotted with the antibodies indicated to the left. FLAG antibody was used to detect ectopically expressed SYT-SSX2 (upper panel). (B) Extracts from pOZ and SYT-SSX2 infectants were immunoblotted with anti-ubiquityl H2A, total H2A and FLAG antibodies. Immunoblotting for alpha-tubulin was used as a loading control. (C) Increased transcription of SYT-SSX2-derepressed polycomb targets. Relative RT-PCR of a subset of SYT-SSX2-upregulated genes also shown to be polycomb-silenced targets [11]. GAPDH served as loading control. D) SYT-SSX2 (upper image), Ring 1B (middle image) and Bmi1 (lower image) occupancy on the NGFR promoter by CHIP-PCR in pOZ vector- and SYT-SSX2-expressing U2-OS cells.

From our aforementioned colocalization experiments, it was evident that at least a subpopulation of Bmi1 depleted in the presence of SYT-SSX2 corresponded to locations that also contained Ring1B (polycomb bodies). We thus wanted to confirm whether the pool of Bmi1 apparently depleted in SYT-SSX2-infectants corresponded to the functional population of Bmi1 normally complexed with Ring1B. The integrity of the Bmi1/Ring1B interaction was therefore assessed by coimmunoprecipitating (co/IP) Bmi1 from SYT-SSX2 and pOZ vector control infectants using anti-Ring1B antibodies. Western blotting of these co/IPs revealed that although equivalent amounts of Ring1B were immunoprecipitated from vector and SYT-SSX2 expressing cells, the quantity of Ring1B-interacting Bmi1 was substantially reduced in SYT-SSX2 infectants (Figure 5A, lower panel).

The pronounced depletion of Bmi1 from the Bmi1/Ring1B complex by SYT-SSX2 raised the possibility that some Bmi1-mediated function related to polycomb silencing may be compromised in the presence of SYT-SSX2. Bmi1 can contribute to polycomb silencing through the facilitation of Ring1B-dependent histone H2A ubiquitination [8]. The loss of Bmi1-Ring1B association in the presence of SYT-SSX2 suggested that a corresponding alteration in histone H2A monoubiquitination might occur as a consequence. To ascertain whether the diminished Bmi1/Ring1B association translated into a loss of H2A K119 ubiquitination, we performed Western blotting analysis to determine the global levels of ubiquityl-H2A in SYT-SSX2-infectants versus controls. Immunoblotting demonstrated that while the levels of total histone H2A and tubulin were unchanged, those of ubiquitinated H2A were dramatically reduced following ectopic expression of SYT-SSX2 (Figure 5B). Taken together, these results demonstrate a general loss of Bmi1-specific histone-modifying function caused by SYT-SSX2-mediated depletion of Bmi1.

### Genomewide analysis of SYT-SSX2-infectants demonstrates reactivation of polycomb target genes

The depletion of Bmi1 and the associated diminution of ubiquityl-H2A provided evidence to support the notion that SYT-SSX2 antagonizes polycomb silencing. Nonetheless, the definitve means to demonstrate a reversion of polycomb-mediated transcription inhibition is to examine whether expression of SYT-SSX2 induces reactivation of polycomb-repressed genes. We performed genomewide analysis of SYT-SSX2-infectants using microarray technology to elucidate the gene expression patterns altered by this oncogene. Hybridization of total RNAs derived from SYT-SSX2 and pOZ vector-infectant transcripts to Affymetrix microarray platforms was performed and transcript changes were expressed as Log_2_ ratios of SYT-SSX2 signal intensity to pOZ control [where the Log_2_ ratio (x) represents 2^x^ fold increases (or decreases) in transcript level]. Only differences of two-fold or greater that recurred in both replicates were designated for further analysis. Of the differentially expressed genes, 123 displayed transcriptional upregulation whereas 33 demonstrated transcriptional downregulation (Microarray of upregulated and downregulated target genes by SYT-SSX2 is available upon request). Gene profiles in SYT-SSX2-expressing cells repeatedly displayed changes in the expression patterns of key proteins involved in cell cycle control, signal transduction, cytoskeletal organization and transcriptional regulation. Many corresponded to previously described SYT-SSX targets (e.g. IGF2) or genes known to be differentially expressed in synovial sarcoma [e.g. EPHB3, TLE3; 25].

In order to determine which of these genes correlated with polycomb targets, the Affymetrix microarray data was compared to another genomewide analysis performed by Lee *et al.*, 2006, [11] in human embryonic cells. These studies, which combined chromatin immunoprecipitation of polycomb-bound promoters with microarray analysis, were designed to determine polycomb-binding regions across the genome. They identified approximately 8% of the human embryonic genome as binding to polycomb complexes and these genes have been designated as putative polycomb targets. In order to compare our SYT-SSX2-differentially expressed genes with this genomewide study, we utilized the Vanderbilt University run, web-based program called WebGestalt (http://bioinfo.vanderbilt.edu/webgestalt/). This program was used to perform bollean operations to determine what percentage of SYT-SSX2-regulated transcripts corresponded to putative polycomb targets. Of the 123 genes upregulated by SYT-SSX2, 33 of them (26%) represent polycomb- repressed genes. These genes are listed in Table 1. This percentage of polycomb reactivated genes represented a greater than 3-fold enrichment over the percentage of genes (8%) that could be theoretically detected by chance alone [11]. These data point towards a trend in polycomb target gene transcript upregulation following ectopic expression of SYT-SSX2.

**Table 1:** Putative polycomb target genes reactivated by SYT-SSX2.

Locus Link ID	Gene Symbol	Gene Name	Log_2_ Ratios^a^	Locus Link ID	Gene Symbol	Gene Name	Log_2_ Ratios
3714	JAG2	Jagged 2	2, 2, **1.9**, **1.9**	387763	LOC387763	Hypothetical LOC387763	1, **1**
6236	RRAD	Ras-related associated with diabetes	3, 2.8, **3.4**, **2.8**	1852	DUSP9	Dual specificity phosphatase 9	1.3, **1.2**
57801	HES4	Hairy and enhancer of split 4	2.4, **2.5**	5979	RET	Ret proto-oncogene	2.4, **2**
9021	SOCS3	Suppressor of cytokine signaling 3	1.2, 1.1, **1.2**, **1.1**	7090	TLE3	Transducin-like enhancer of split 3	1.3, **1.3**
4804	NGFR	Nerve growth factor receptor	5.4, **5.4**	9244	CRLF1	Cytokine receptor-like factor 1	3, **2.9**
2049	EPHB3	EPH receptor B3	1.2, **1.2**	54855	FAM46C	Family with sequence similarity 46, member C	3.3, 1.6, **3.3**, **1.3**
8913	CACNA1G	Calcium channel, alpha 1G	1.8, **2**	10018	BCL2L11	BCL2-like 11	1.2, **1.3**
28514	DLL1	Delta-like 1	5.3, **5.4**	30812	SOX8	SRY-box 8	6.5, **6.3**
23345	SYNE1	Spectrin repeat containing, nuclear envelope 1	1.3, **1.2**	4616	GADD45B	Growth arrest and DNA damage-inducible 45	1.2, 1.1, 1, **1.1**, **1.1**, **1**
284207	METRNL	Meteorin	2, **2.2**	8535	CBX4	Chromobox homolog 4	1.3, **1.4**
5155	PDGFB	Platelet-derived growth factor beta	4.1, 1.9, **3.4**, **1.7**	131583	FAM43A	Family with sequence similarity 43, member A	3.3, **3.3**
4772	NFATC1	Nuclear factor of activated T-cells	1.1, 1, **1.2**, **1.1**	6299	SALL1	Sal-like 1	1.2, **1.2**
9123	SLC16A3	Solute carrier family 16, member 3	2.7, 2.6, **2.8**, **2.5**	2643	GCH1	GTP cyclohydrolase 1	1.4, **1.3**
10966	RAB40B	RAB40B, member RAS family	1.1, **1**	4487	MSX1	Msh homeobox 1	1.4, **1.2**
7107	GPR137B	G-protein receptor 137B	1.1, **1**	4062	LY6H	Lymphocyte antigen 6 complex, l ocus H	1.9, **1**
10568	SLC34A2	Solute carrier famiy 34, member 2	4.1, **4**	816	CAMK2B	Calcium/calmodulin-dependent kinase 2	4.3, **4.2**
164633	CABP7	Calcium binding protein 7	4.1, 4.1				

a Nonbolded log_2_ ratios represent hits in microarray replicate 1 **bolded** log_2_ ratios represent replicate 2.

### Antagonism of polycomb by SYT-SSX2

To confirm the overlap between the SYT-SSX2 microarray expression profile and the reported polycomb gene targets [11], upregulated transcription of a representative group of shared targets was validated by relative RT-PCR (Figure 5C). The effect of SYT-SSX2 on the Ring1B/Bmi1 complex was further characterized on a representative promoter- that of the NGFR gene- by chromatin immuno-precipitation (CHIP). Our choice of an appropriate target for such studies was based on the persistent upregulation of NGFR in all the microarrays performed on SYT-SSX2-expressing mesenchymal cells (Figure 5C and data not shown). Our CHIP analyses confirmed that the NGFR promoter is indeed a direct target of SYT-SSX2 (Figure 5D, upper panel). They also established that while Ring 1B is present at the promoter in vector control- and SYT-SSX2 expressants (Figure 5D, middle panel), Bmi1 was no longer detected on the NGFR region in SYT-SSX2 cells (Figure 5D, lower panel). These results are consistent with the Ring 1B- and Bmi1- SYT-SSX2 colocalization data (Figure 1B) and Bmi1 diminution upon SYT-SSX2 expression.

In summary, the SYT-SSX2 oncogene appears to influence gene-expression programs through its association with and subsequent antagonism of polycomb components in mesenchymal cells. This function likely constitutes part of its transforming activity in synovial sarcoma.

## Discussion

In this study, we demonstrate a consistent and significant depletion of the polycomb complex protein Bmi1 following *de novo* expression of the SYT-SSX2 oncogene. This attenuation of Bmi1 appears to be mediated at the level of protein stability, although the specific mechanism by which SYT-SSX2 promotes Bmi1 degradation awaits further investigation. The expected outcome of Bmi1 degradation in the presence of SYT-SSX2 is the alteration of at least certain aspects of polycomb silencing function. Indeed, evidence exists to corroborate the expected impairment of polycomb gene regulation. First, we demonstrated the predicted loss of global histone H2A monoubiquitination resulting from SYT-SSX2-mediated Bmi1 depletion. This effect most likely stems from a decrease in Bmi1 facilitation of Ring1B E3-ligase activity towards H2A, as the population of depleted Bmi1 corresponded largely to the pool that interacted with Ring1B. Whether the reduction in Bmi1 alters the positioning of Ring1B relative to H2A and/or affects the polyubiquitination status of Ring1B remains to be elucidated. To date, this is the only specific polycomb function that has been shown to be compromised by SYT-SSX2, suggesting that only a subset of polycomb target genes may be affected by the chimera.

In addition, we presented evidence to suggest that the Bmi1 depletion and histone H2A hypoubiquitination correlated with the reactivation of polycomb-repressed genes. To demonstrate this derepression, we adopted a genomewide approach that relied on microarray analysis to determine which transcripts were induced by SYT-SSX2. The output from these analyses were compared with another study whereby polycomb target promoters were identified utilizing a combination of chromatin immunoprecipitation and microarray analysis. One important limitation of these comparison studies is that the genomewide analysis conducted by Lee *et al.* (2006), as well as others, were performed in the context of embryonic stem (ES) cells. However, studies performed on a prototypic polycomb target promoter –the NGFR gene- corroborated our central observation of Bmi1 attenuation by SYT-SSX2. Further chromatin studies in U2-OS and other mesenchymal cells are ongoing.

Although the influence of indirect effects cannot be discounted and unlisted genes may be down-regulated to undetectable levels, the microarray results support the notion that SYT-SSX2 may predominantly activate rather than suppress genes. Notably, a large number (more than half) of the 33 derepressed polycomb targets were also upregulated in the microarrays of the SYT-SSX2-derived murine synovial sarcomas as well as human synovial sarcoma tissues [23], [32], [33], [34], [35]. The overlap includes either the same factors such as Jagged2 (JAG2), Delta-like 1 (DLL1), Sal-like 1 (SALL1), dual specificity phosphatase 9 (DUSP9), nerve growth factor receptor (NGFR), ephrin B3 receptor (EPHB3), suppressor of cytokine signaling 3 (SOC3), Msh-homeobox 1 (MSX1), chromobox homolog 4 (CBX4), BCL2-Like11 (BCL2L11), cytokine receptor-like factor 1 (CRLF1), member RAS family (RAB40), solute carrier family 16 (SLC16), nuclear factor of activated T-cells (NFATC1), or members of the same signaling families such as JAG1, SALL2, MSX2, transducin-like enhancer of split 3 (TLE3), G-protein receptors (GPR), ephrin receptors (EPHB1,2,4), NFATC4, CBX2,5, and platelet-derived growth factor (PDGF). Several of the polycomb-silenced transcripts upregulated by SYT-SSX2 corresponded to proteins that are implicated in tumor formation and progression. Several members of the Notch signaling pathway, including *DLL1*, *JAG2* and *HES4*, displayed increased steady state mRNA in the presence of SYT-SSX2. Activating mutations of Notch signaling receptors or overexpression of the Notch ligands (DLL, JAG) have been observed in hematological, breast, cervical, and intestinal tumors [38]. Increased Notch signaling can promote cellular transformation, epithelial-to-mesenchymal transition and tumor vascularization in the appropriate context. Another gene, *EphB3*, corresponds to a receptor of the ephrin signaling pathway, a cascade whose deregulation contributes to tumor cell shape, adhesion, migration and angiogenesis [7]. Finally, *PDGF-B* corresponds to a ligand in the platelet derived growth factor pathway that can mediate tumor migration and capillary network formation. Reactivation of these genes by SYT-SSX2 could therefore ultimately facilitate tumorigenesis and/or tumor progression in synovial sarcoma.

The finding that a reputed oncogene can suppress certain aspects of polycomb silencing was somewhat unexpected. The majority of work examining the role of polycomb silencing in cancer has established that oncogenicity is often associated with a polycomb gain of function. However, several lines of evidence, including the observed reactivation of the polycomb target *Hox* genes [19], [21] and downregulation of some polycomb proteins in limited cancers [16], have suggested that polycomb derepression can equate to tumorigenesis in the appropriate context. In addition, the transforming activity attained by a loss-of-function mutant of the polycomb subunit PC2 provides more direct evidence that polycomb silencing can also be impaired in cancer. Recently, a landmark study also demonstrated that a consistent decrease in global ubiquityl-histone H2A occurs in prostate cancer, suggesting that H2A hypoubiquitination may serve as a tumor marker in this disease [21]. The significance of the work presented in the current study lies in the novel demonstration of disrupted polycomb silencing caused by an oncogene.

A crucial and precarious balance in Polycomb function is needed for maintaining the homeostasis of the cell. This balance in polycomb gene silencing is mediated by processes including histone H2A ubiquitination, chromatin compaction, RNA polymerase II inhibition, histone hypoacetylation and DNA methylation. When any of these functions are perturbed through a gain of polycomb silencing, such as when polycomb components are overexpressed, tumor-promoting phenotypes are induced through the repression of tumor suppressors. However, a shift towards impairment of certain polycomb silencing functions can also promote cancer through the reactivation of oncogenic genes. In this regard, it is important to emphasize that many polycomb gene targets are overexpressed in cancer, including members of the Wnt, Notch and ephrin signaling pathways [9]–[11]. Although it has yet to be determined, a possible mechanism explaining aberrant signaling through these pathways in cancer may reside in the suppression of polycomb silencing.

Based on the available evidence, we believe that SYT-SSX2 protein is recruited to a subset of polycomb repressive complexes within the nucleus via direct and/or indirect interactions with polycomb components or other co-residing chromatin modifiers. This SYT-SSX2 recruitment promotes the displacement and/or degradation of Bmi1 protein, which alters normal Bmi1-mediated functions, such as histone H2A monoubiquitination. In addition, the SYT-SSX2 chimera, through physical interactions [29], [30], may recruit both the SWI/SNF chromatin remodeling complex and p300 acetyltransferase to this polycomb-repressed chromatin. The aberrant targeting of these proteins may then promote changes that culminate in the transcription of genes that were once silenced by polycomb complexes. The protein products of these upregulated genes could then ultimately contribute to some aspect of synovial sarcoma pathogenesis.

Taken together, these studies provide an interesting insight into the molecular function of SYT-SSX2. When carried further, these analyses of chromatin deregulation by the fusion protein will be beneficial for targeting synovial sarcoma. Moreover, they will enhance our understanding regarding the regulation of polycomb components.

## Materials and Methods

### Cells and Reagents

The U2OS osteosarcoma cell line was maintained in Dulbecco's Modified Eagle Medium (DMEM) supplemented with 10% fetal bovine serum (FBS). Antibodies for Western blotting and immunofluoresence included anti-Bmi1 (Upstate; Lake Placid, NY), anti-Ring1B (MBL; Woburn, MA), anti-YY1 (Santa Cruz Biotechnology; Santa Cruz, CA), anti-tubulin (Sigma; St. Louis, MO), anti-ubiquitinated H2A (Upstate; Lake Placid, NY), anti-H2A (Upstate; Lake Placid, NY) anti-HA (Sigma; St. Louis, MO) and anti-FLAG (Sigma; St. Louis, MO). The SSX2-specific polyclonal antibody and the SV11 monoclonal antibody were described in Pretto *et al*, 2006 [**39**].

### Plasmids

The SYT, SYTdel8, SYT-SSX2 cDNAs were inserted into the pOZ retroviral construct (a gift from P. Nakatani) as described previously [39]. Construction of pGST-SYT-SSX2 was previously described [29]. The LZRS-Bmi12PY-IRES-GFP and pGST-Bmi1 vectors were kind gifts from M. Lohuizen. The pCS2^+^-Ring1B vector for *in vitro* translation of Ring1B was a gift from A. Ciechanover. For construction of SYT-SSX2 deletion mutants, designated SYT-SSX2del1-del9, site directed mutagenesis was performed using pOZ-SYT-SSX2 as a template. Primers utilized in the mutagenesis reactions were as follows: SYTSSXDel1F-5′-CCTTATGGATATGACCAGG-TGCCAGAAGCATCTGGC-3′; SYTSSXDel1R-5′-GCCAGATGCTTCTGGCACCT-GGTCATATCCATAAGG-3′; SYTSSXDel2F-5′-GGAAATGATTCGGAGGAAAC-TACCTCTGAGAAGATT-3′; SYTSSXDel2R-5′-AATCTTCTCAGAGGTAGTTTCC-TCCGAATCATTTCC-3′; SYTSSXDel3F-5′-TGCCCCCCGGGAAAACCACACAGA-CTGCGTGAGAGA-3′; SYTSSXDel3R-5′-TCTCTCACGCAGTCTGTGTGGTTTTC-CCGGGGGGCA-3′; SYTSSXDel4F-5′-GAACATGCCTGGACCGGCCGCTGGAGG-AGACTA-3′; SYTSSXDel4R-5′-TAGTCTCCTCCAGCGGCCGGTCCAGGCATGTT-C-3′; SYT-SSXDel5F-5′-TGCCCCCCGGGAAAACCAGGACCCAAAAGGGGGGA-AC-3′; SYT-SSXDel5R-5′-GTTCCCCCCTTTTGGGTCCTGGTTTTCCCGGGGGG-CA-3′; SYT-SSXDel6F-5′-TCTCTCACGCAGTCTGTGAGATCTCTCGTGAATCTT-3′; SYTSSX-Del6R-5′-TCTCTCACGCAGTCTGTGAGATCTCTCGTGAATCTT-3′; SYTSSXDel-7F-5′-TCTGGACCCAAAAGGGGGAGAAAACAGCTGGTGATT-3′; SYTSSXDel7R-5′-AATCACCAGCTGTTTTCTCCCCCTTTTGGTCCAGA-3′; SYTSSXDel8F-5′-GGGAACATGCCTGGACCATTTATGAAGAGATCAGC-3′; SYTSSXDel8R-5′-GCTGATCTCTTCATAAATGGTCCAGGCATGTTCCC-3′; SYTSSXDel9F-5′-GAGAGAAAACAGCTGGTGGCGGCCGCTGGAGGAGAC-3′; SYTSSXDel9R-5′-GTCTCCTCC AGCGGCCGCCACCAGCTGTTTTCTCTC-3′.

### Retroviral Transduction of U2OS Cells

The SYT, SYTdel8, SYT-SSX2 cDNAs were inserted into the pOZ retroviral vector and the infections were conducted as described previously [39].

### RT-PCR

For RT-PCR analysis, total RNA was first extracted from retrovirally-infected cells using the RNAeasy Miniprep Kit (Qiagen). 1 µg of total RNA was subjected to reverse transcription reaction using the Superscript II Reverse Transcription System (Invitrogen). A 1 µl volume of the cDNA reaction was amplified using PCR with the following specific primers, BMI1-F: 5′-GGTACTTCATTGATGCCACAAC-3′, BMI1-R: 5′-CTGCTGGGCATCGTAAGTATC-3′, NGFR-F: 5′-GGCACCTCCAGAACAA-GACCTC-3′, NGFR-R: 5′-ACAGGGATGAGGTTGTCGGTG-3′, RRAD-F: 5′-TTTA-CAAGGTGCTGCTGCTGGG-3′, RRAD-R: 5′-TGCCGCTGATGTCTCAATGAAC-3′, RET-F: 5′-GGATTTCGGCTTGTCCCGAG-3′, RET-R: 5′-CCATGTGGAAGGGAGG-GCTC-3′, PDGF-B F: 5′-GATCCGCTCCTTTGATGATC-3′, PDGF-B R: 5′-GTCTCA-CACTTGCATGCCAG-3′, HES4-F: 5′-CACCGCAAGTCCTCCAAG-3′, HES4-R: 5′-TCACCTCCGCCAGACACT-3′. IGF2-F: 5′-GAAGTCGATGCTGGTGCTTCT-3′, IGF2-R: 5′-TGAACGCCTCGAGCTCCTTG-3′, DLL1-F: 5′-GTTCGAACTGAAGCT-GCAGGA-3′, DLL1-R: 5′-AGAATCTGTGTGGAGAGCTTC-3′, JAG2-F: 5′-AAGAC-CTGAACTACTGTGGCA, JAG2-R: 5′-GCATGGCTTCCCTTCACACT-3′, EPHB3-F: 5′-GGTGACGTCTGAGCTGGCATG-3′, EPHB3-R: 5′-TCATCTGGCGCAATGGTG-TCC-3′. GAPDH loading control was amplified using the following primers, GAPDH-F: 5′-TGATGACATCAAGAAGGTGGTGAAG-3′ and GAPDH-R: 5′-TCCTTGGAGG-CCATGTGGGCCAT-3′. Reaction conditions for PCR amplification are as follows: 1 cycle of 4 min. at 94°C; 32 cycles of 1 min. at 94°C, 1 min. at 56°C and 1 min. at 72°C; 1 cycle of 10 min. at 72°C.

### *In vitro* Binding Assays

The generation of GST, GST-p300-CH3, GST-Bmi1 and GST-SYT-SSX2 lysates was performed as previously described [28]. *In vitro* translation of SYT-SSX2 and Ring1B was carried out using the coupled TnT rabbit reticulocyte *in vitro* transcription/translation system (Promega Co; Madison, WI). Transcription/translation was carried out in the presence of SP6 RNA polymerase and 20 µCi S^35^-methionine. Binding reactions of *in vitro* translated proteins and GST-fused proteins were performed in the presence of binding buffer (20 mM Tris pH 8.0, 100 mM NaCl, 0.1% NP-40, 1 mM DTT) for 1 hr. at 4°C. Beads were washed in ice-cold binding buffer 3 times, then boiled in Laemmli sample buffer. Reactions were electrophoresed on a 10% SDS-polyacrylamide gel, dried and exposed to x-ray film for autoradiography.

### Indirect Immunofluorescence

For indirect immunofluorescence studies, coverslips were fixed in 3% paraformaldehyde/ 2% sucrose, washed with PBS and permeabilized in 0.2% Triton X-100. Cells were blocked in 3% goat serum and incubated with anti-Bmi1 (1∶100), anti-Ring1B (1∶100) and anti-HA (1∶200) antibodies for 2 hrs, then with Alexa-conjugated secondary antibodies (Molecular Probes; Eugene, OR) for 30 min. Cells were visualized using a Zeiss (Axioplan 2) fluorescence microscope.

### Microarray Analysis

Total RNA was extracted from infected NIH3T3 cells using the RNAeasy Miniprep Kit (Qiagen; Valencia, CA) according to the manufacturer's instructions. For microarray experiments, RNA was submitted to the Vanderbilt Microarray Shared Resource Facility for hybridization with Affymetrix Human Genome U133 GeneChip arrays. Gene expression intensity changes were expressed as Log_2_ ratios of SYT-SSX2 signal intensity to pOZ control [where the Log_2_ ratio (x) represents 2^x^ fold increases (or decreases) in transcript level]. Analysis of microarray data was carried out by the WebGestalt Program, maintained by Vanderbilt University and available online at http://bioinfo.vanderbilt.edu/webgestalt/.

### Lysis and Immunoprecipitations

To generate extracts for Western blotting analysis of ubiquitinated H2A, infected cells were lysed and boiled directly in Laemmli sample buffer. Lysate preparation for immunoprecipitation and other Western blotting procedures were performed in IP buffer (50 mM Tris pH 8.0, 250 mM NaCl, 0.5% NP-40 and protease inhibitor cocktail). Lysates used for immunoprecipitation were incubated with primary antibodies (all at 1 µg/mL final concentration) for 4 hrs at 4°C. Samples were then incubated with Protein G-sepharose (Amersham Biosciences; Uppsala, Sweden) for 30 min at 4°C. Immunoprecipitates were then washed in IP buffer and boiled in Laemmli sample buffer for Western blot analysis.

### Pulse Chase Experiments, Peptide Blocking and Regression Analysis

U2OS cells that stably expressed 2PY-Bmi1 were infected with pOZ and SYT-SSX2 vectors as described earlier. At 48 hrs post-infection, cells (2×10^5^ cells per timepoint for each experimental condition) were starved in Methionine-minus DMEM medium (containing 5% dialyzed fetal bovine serum) for 1 hr at 37°C. Cells were then labeled with 500 µCi of S^35^ labeled Methionine/Cystine diluted in Methionine-minus medium (containing 5% dialyzed fetal bovine serum) for 1 hr at 37°C. Cells were then chased at indicated time-points with Methionine-positive DMEM supplemented with 10% fetal bovine serum. Cells from each timepoint were processed and immunoprecipitated with anti-polyoma tag antibody (1 µg/mL) as described before. Immunoprecipitates were run on 10% polyacrylamide gels, which were subsequently dried and exposed to x-ray film for autoradiography. Immunoprecipitated 2PY-Bmi1 was quantitated using densitometry computer software (FluorChem 9800; Alpha Innotech, San Leandro, CA). To confirm the correct identity of the immunoprecipitated 2PY-Bmi1 species, 1×10^6^ cells were labeled overnight with S^35^ labeled Methionine/Cystine. Cells were immunoprecipitated with anti-polyoma antibodies as before, except that the following conditions were included: naïve U2OS cells, no antibody control, preclearing of lysate with Sepharose beads (30 min. at 4°C) and overnight preincubation of polyoma antibody with polyoma epitope tag peptide (20 µg/mL; Covance, Emeryville, CA). For the regression analysis, densitometry values for pOZ and SYT-SSX2 were plotted on GraphPad Prism software (GraphPad Software Inc., La Jolla, CA) for linear regression analysis and half-life calculations. Half-lives were assessed by determining the unpaired half-life time-point values from a relative intensity value of 0.5.

### Chromatin Immuno-Precipitation

CHIP was performed on pOZ- and SYT-SSX2-derived nuclear lysates according to the protocol described by Boyer et al. [40]. 2×10^7^ cells were used for each IP reaction. After DNA precipitation, PCR of the NGFR promoter region was performed with the following primers: NGFR-forward: 5′-GCAGTTAGGGAGCAAGGCTCC-3′ and NGFR-reverse: 5′-GGTGGGA-AGCAGAGGCAAAGG-3′. SYT-SSX2 was IP'ed with the SSX2-specific antibody described in Pretto et al. [39]. For Ring1B and Bmi1 CHIP, the MBL and Upstate antibodies described above were used, respectively.

## Supporting Information

Figure S1 In vitro binding assays
(0.54 MB DOC)

Figure S2 SYT-SSX2 does not alter Bmi1 mRNA levels
(0.10 MB DOC)
