# Supplementary material for: The Synovial Sarcoma-Associated SYT-SSX2 Oncogene Antagonizes the Polycomb Complex Protein Bmi1
Source: PLoS One. 2009 Apr 1;4(4):e5060. doi: 10.1371/journal.pone.0005060 (PMC2659801; doi:10.1371/journal.pone.0005060)
Supplement: Figure S1 — In vitro binding assays (0.54 MB DOC) [file pone.0005060.s001.doc]

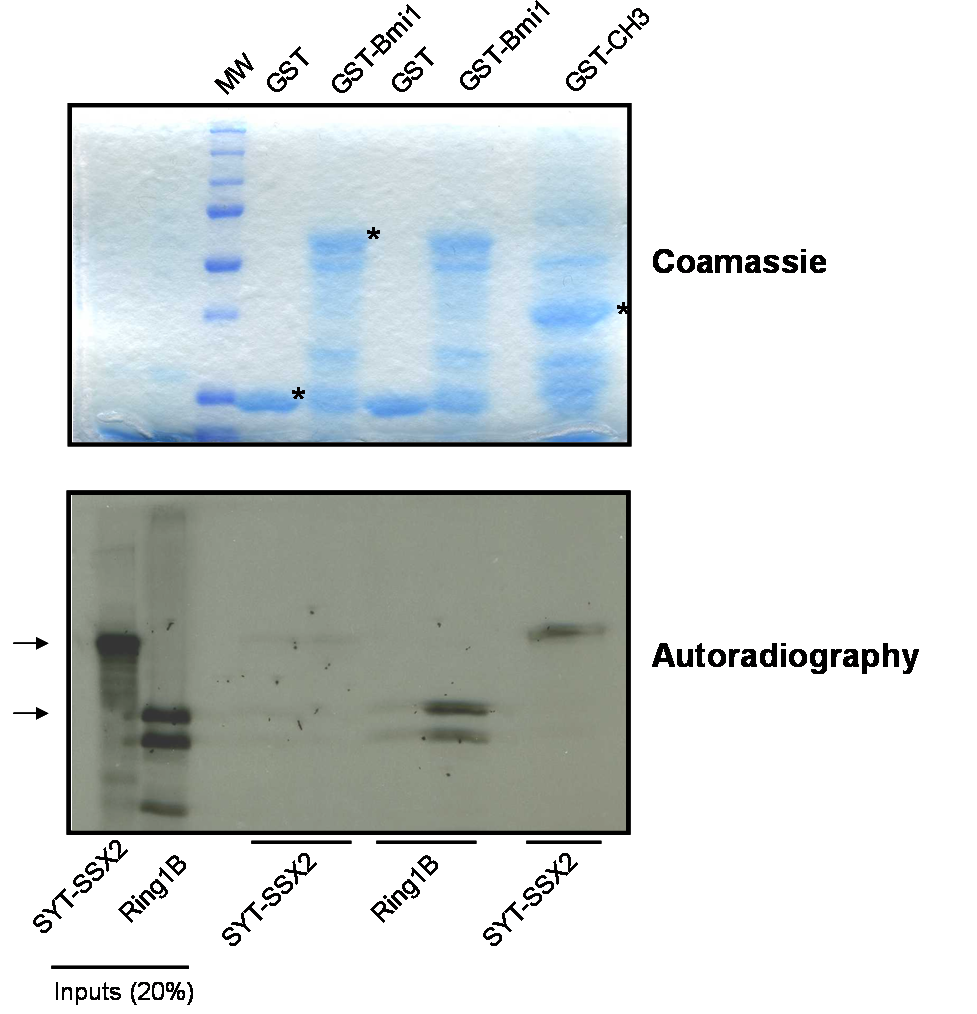


**Supplemental Figure 1. *In vitro* binding assays**. SYT-SSX2 does not bind directly to Bmi1. *In vitro* translated SYT-SSX2 or Ring1B was incubated with either GST, GST-p300CH3 or GST-Bmi1. Binding reactions were pulled down and detected by coomassie staining and autoradiography. Labels above the coamassie gel represent the GST-protein used in each condition, while astericks denote the full length GST-fused protein (MW=molecular weight marker). Labels below the autoradiography gel indicate the *in vitro* translated proteins (SYT-SSX2 or Ring1B) with which the GST proteins were incubated. The top arrow depicts full length *in vitro* translated SYT-SSX2, while the bottom arrow indicates Ring1B.
