# Supplementary material for: The Synovial Sarcoma-Associated SYT-SSX2 Oncogene Antagonizes the Polycomb Complex Protein Bmi1
Source: PLoS One. 2009 Apr 1;4(4):e5060. doi: 10.1371/journal.pone.0005060 (PMC2659801; doi:10.1371/journal.pone.0005060)
Supplement: Figure S2 — SYT-SSX2 does not alter Bmi1 mRNA levels (0.10 MB DOC) [file pone.0005060.s002.doc]

**Relative RT-PCR**

##
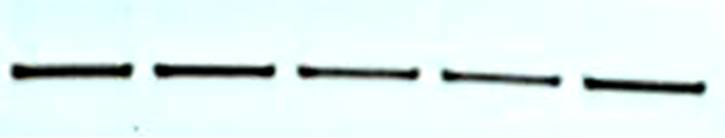

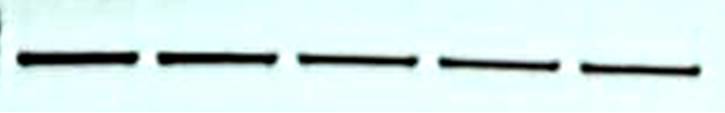


**POZ SYT SYT SYT SSX2**

**-SSX2 del8**

## Supplemental Figure 2. SYT-SSX2 does not alter Bmi1 mRNA levels. Total RNA was extracted from SYT-SSX2 or pOZ backbone vector-infected U2OS cells. Relative RT-PCR was performed using primers specific to Bmi1 and to GAPDH.
